# Glycemic control and association with diabetes-related distress, self-management behavior, financial toxicity, and cost-related non-adherence: a mixed-methods study

**DOI:** 10.3389/fendo.2026.1857675

**Published:** 2026-06-05

**Authors:** Abdulfatai Olamilekan Babaita, Iyabo Yewande Ademuyiwa, Michiko Moriyama

**Affiliations:** 1 Graduate School of Biomedical and Health Sciences, Program of Health Sciences, Hiroshima University, Hiroshima, Japan; 2 Department of Nursing Science, Faculty of Health Professions, University of Lagos, Lagos, Nigeria; 3 Division of Nursing Science, Graduate School of Biomedical and Health Sciences, Hiroshima University, Hiroshima, Japan

**Keywords:** determinants, diabetes mellitus, type 2, financial stress, glycated hemoglobin, glycemic control

## Abstract

**Background:**

The prevalence of diabetes is a growing global health concern. Currently, Nigeria has the highest burden of diabetes in sub-Saharan Africa, and this burden is projected to increase by over 100% by 2050. In a lower-middle-income country, a comprehensive understanding of the emotional burden, behavioral factors, and financial determinants of glycemic control is imperative.

**Methods:**

An explanatory sequential study design was conducted among patients with T2DM attending follow-up visits at three secondary-level hospitals. For the cross-sectional strand (n = 355), the Diabetes Distress Scale (DDS), Diabetes Self-Management Questionnaire (DSMQ-R), Financial Toxicity, and Cost-related Non-adherence questionnaires were used. Long-term glycemic control (HbA1c) was estimated using the Clover A1c system (Infopia). Moreover, semi-structured interviews (n = 19) were conducted to further explore the effect of psychosocial, behavioral, and financial factors on glycemic control.

**Results:**

The mean HbA1c was 7.04% (SD: 2.2), and 43% (n= 152) of the respondents had HbA1c ≥7%. The percentage of participants with diabetes-related distress, poor self-management, worse financial toxicity, and the practice of cost-related non-adherence was 32%, 56%, 54%, and 48.3%, respectively. Although poor self-management (β: 2.02; CI: 1.18 – 3.45) and diabetes-related distress (β: 2.13; CI: 1.22 – 3.72) were significantly associated with poor glycemic control, financial toxicity and cost-related non-adherence were not significantly associated with glycemic control. Younger age (<55 years), use of multiple antidiabetic medications, insulin use, and lack of access to an endocrinologist were significant covariates associated with poor glycemic control. In the qualitative interview, the themes extracted for diabetes-related distress and financial toxicity in the good and poor glycemic control groups were relatively similar. However, there was a considerable difference in the diabetes self-management behavior; participants with poor glycemic control had (1) ‘difficulty adapting to lifestyle changes’ and (2) ‘medication non-adherence due to fear of after effects from prolonged use or polypharmacy’.

**Conclusion:**

Approximately half of the participants across the study sites had poor glycemic control. Poor self-management, diabetes-related distress, the type of treatment regimen, and lack of access to an endocrinologist are independent determinants of poor glycemic control. Diabetes education grounded in behavioral modification strategies and psychological support should be a routine practice. Moreover, the inclusion of trained primary care physicians and endocrinologists in care is imperative.

## 1 Background

Diabetes mellitus is a major public health concern that affects 11.1% of the world’s adult population (589 million adults aged 20-79 years). It is one of the fastest-growing global health emergencies of the twenty-first century; by 2050, the prevalence is projected to increase by 45% (853 million). Moreover, type 2 diabetes, which accounts for 90% of diabetes cases, is the eighth-leading cause of disease burden and is projected to become the second-leading cause by 2050 (1, 2).

Despite the low prevalence of diabetes in Africa, the region is expected to experience a 142% increase by 2050; this is the highest projection among the International Diabetes Federation (IDF) regions". "Although, by region, Africa has the lowest prevalence of diabetes, the region is expected to experience a 142% increase by 2050; this is the highest projection among the International Diabetes Federation (IDF) regions (3). In sub-Saharan Africa, Nigeria has the highest number of people with diabetes, and this number is expected to double by 2050 (1). Achieving good glycemic control is the mainstay of diabetes treatment (4). However, studies across Nigeria have reported a high prevalence of poor glycemic control (5–8). The failure to manage hyperglycemia, which is the hallmark of diabetes, becomes a risk factor for several health conditions and chronic complications, including retinopathy, nephropathy, neuropathy, coronary artery disease, peripheral artery disease, and stroke (4, 9). These complications are chronic and add to the already substantial disease burden experienced by patients. For instance, in a multicenter study in Nigeria, 13.8% of admissions to the medical ward were diabetes-related, and diabetic foot ulcers accounted for 24.9% of admissions; the mortality rate after admission was 20.5% (10). A population-based cohort study (11) and the UK Prospective Diabetes Study (UKPDS) (12) showed that intensive glycemic control early in the disease course reduced the majority of microvascular complications.

The rising morbidity and mortality associated with diabetes are linked to poor control of hyperglycemia; however, the contributing factors are multifaceted. Some studies have reported sociodemographic and clinical factors, such as age, sex, level of education, income, obesity, and treatment type, as determinants of poor glycemic control (13, 14). Likewise, factors related to patients and healthcare service delivery have been identified as contributors to glycemic control (15). Other studies have found that aspects of self-management behavior, such as poor medication adherence, inadequate physical activity, limited self-management knowledge, psychosocial factors, and diabetes self-efficacy, are associated with glycemic control (16–18).

In Nigeria, studies on the determinants of glycemic control have focused primarily on sociodemographic and clinical factors. Although studies have examined the level of self-management behavior and diabetes-related distress (5, 6), there is limited understanding of their effect on glycemic control. Likewise, in a lower-middle-income country with inefficient healthcare insurance, there is a paucity of evidence regarding the impact of financial hardship on glycemic control. In Ipingbemi et al. (19), financial constraints were the primary reason cited for medication non-adherence and a leading reason for non-adherence to dietary recommendations.

Regarding financial hardship, the concept of financial toxicity is well established in the cancer literature and gaining relevance in the diabetic population (20, 21). It describes the economic burden related to the cost of medical care, and the resulting financial strain has a detrimental effect on an individual’s wellbeing and quality of life (22, 23). The major cause of financial distress is the out-of-pocket expenses patients incur during the course of management of the disease (23). Likewise, cost-related non-adherence has been linked to poorer health outcomes. A primary barrier to medication adherence is the high cost of medication, which patients pay for out of pocket (24). For people managing chronic conditions, such as diabetes, the ongoing need to buy medication may subsequently lead to engagement in maladaptive cost-coping behaviors that lead to medication non-adherence (21, 25, 26). These two measures of financial hardship could further expand the understanding of the impact of socioeconomic determinants of health, beyond low education and low income, on glycemic control. In a scoping review, people with high out-of-pocket expenditure, unstable employment, and low income were more likely to report cost-related non-adherence (27). Based on available evidence in the elderly population with diabetes, measures of financial hardship, such as difficulty paying bills (0.25, [95%CI 0.14 - 0.35]) and medication cost non-adherence (0.17, [95%CI 0.03 - 0.31]), were significantly associated with HbA1c (28).

Given that the Nigerian healthcare system still operates within a traditional acute-care model for the management of chronic conditions, in which behavioral and psychosocial support for patients is often overlooked in clinical settings, and considering the economic realities of a lower-middle-income country, exploring the psychosocial, behavioral, and financial determinants of glycemic control is imperative. Therefore, in addition to socioeconomic factors, this study aimed to investigate the level of diabetes-related distress, self-management practices, financial toxicity, and cost-related non-adherence, and their relationship with glycated hemoglobin (HbA1c). We hypothesized that (1) participants with diabetes-related distress, poor self-management practices, worse financial toxicity, and cost-related non-adherence are more likely to have poor glycemic control, and (2) participants who engage in cost-coping behaviors will have lower financial toxicity score than those who do not engage in cost-coping behaviors.

## 2 Methods

### 2.1 Study design

This study is an analytical cross-sectional study conducted among people living with type 2 diabetes attending the outpatient clinics of three secondary-level general hospitals in Lagos state, Nigeria. For the quantitative strand, a cross-sectional design was employed to explore the behavioral, financial, and psychosocial factors associated with poor glycemic control. Subsequently, semi-structured interviews were conducted using a purposefully selected subsample of the participants based on maximum variation in glycemic control and survey questionnaire responses to expand understanding of the quantitative results. This study was conducted between January 2025 and March 2025.

### 2.2 Quantitative data collection and analysis

#### 2.2.1 Sample

The sample was calculated on the basis of 10 events per variable using the formula: N = 10k/p. If the number of events (i.e., poor glycemic control) is at least 10 times greater than the number of independent variables, considering 17 independent variables (including all dummy variables), the number of events should exceed 170. Assuming a proportion of poor glycemic control of 50%, the sample size required would be 170/0.50 = 340. With a 10% non-response rate, the total sample size required would be 374.

Using convenience sampling, participants were eligible if (1) were 18 years of age or older, (2) had type 2 diabetes, (3) were receiving medication management, and (4) had HbA1c data available within the previous 3 months or, if not, agreed to a simple test performed by the research assistant. Participants were excluded if they had (1) obvious cognitive impairment, (2) psychiatric disorders, or (3) no drug prescriptions.

#### 2.2.2 Setting

Three secondary-level healthcare facilities under the Lagos State Health Service Commission (LSHSC), one from each of the three senatorial districts in Lagos State, were purposefully selected. The medical outpatient departments included endocrinology clinics staffed by endocrinologists (except in one facility) and primary care physicians who served as care providers. Approximately 30 to 40 patients attended the clinics for follow-up visits daily. Secondary-level facilities were selected because of the presence of general practitioners, who constitute the largest proportion of the diabetes care workforce in Nigeria, and because of the preference of the Nigerian population for accessing secondary-level facilities for medical care.

#### 2.2.3 Data collection

Before the commencement of the study, training on the use of the questionnaires was provided to the research assistants (RAs). On clinic days at the respective hospitals, patients were either approached in the waiting area or encouraged to participate by nurses and physicians. After the study was explained and informed consent was obtained, participants completed the questionnaires either using Microsoft Forms on iPads or in paper format. When necessary, the RAs assisted participants in completing the questionnaires. After completion, a blood sample (0.004 mL) was collected from the fingertip by a medical laboratory scientist intern, and the HbA1c test was performed using the Clover A1c Analyzer (Infopia, Korea). The participants received their test results and hand sanitizer as a token of appreciation.

#### 2.2.4 Instrument

##### 2.2.4.1 Demographic and clinical characteristics

The demographic and clinical variables collected included age, sex, weight, height, blood pressure, monthly income, household income, educational attainment, employment status or occupation, health insurance status, ethnicity, religion, number of antidiabetic medications, marital status, duration of illness, comorbidities, and body mass index (BMI).

##### 2.2.4.2 HbA1c

HbA1c was used as the biomarker of long-term glycemic control. The Clover A1c Analyzer (Infopia, Korea), which uses the boronate affinity method, was used to estimate participants’ HbA1c levels. The analyzer is certified by the National Glycated Heamoglobin Standardization Program (NGSP), USA, and the International Federation of Clinical Chemistry (IFCC). Based on the recommendations of the American Diabetes Association (4), HbA1c levels < 7% and ≥ 7% were classified as good glycemic control and poor glycemic control, respectively.

##### 2.2.4.3 Diabetes Distress Scale

The 17-item Diabetes Distress Scale (DDS), developed and validated by Polonsky et al. (29), was used to measure diabetes-related distress. The scale has four dimensions: emotional burden (EB), regimen distress (RD), interpersonal distress (ID), and physician distress (PD). Items are rated using a 6-point Likert scale (1 = ‘not a problem’ to 6 ‘a very serious problem’). The score on the scale ranges from 17 to 102. To compute the average score, the sum of the item scores is divided by the number of items on the scale (17). This approach can also be applied to the subscales. Average scores of <2.0, 2.0 – 2.9, and ≥3.0 on the scale and subscales were considered no distress, moderate distress, and high distress, respectively. A score of 2.0 or higher reflects the presence of diabetes-related distress. As reported by Polonsky et al., the Cronbach’s alpha values for the total scale and subscales were as follows: total: 0.93; EB: 0.88; PD: 0.88; RD: 0.90; and ID: 0.88. In the current study, the Cronbach’s alpha values were: total: 0.87; EB: 0.80; PD: 0.71; RD: 0.74; and ID: 0.87.

##### 2.2.4.4 Diabetes Self-Management Questionnaire–Revised

The Diabetes Self-Management Questionnaire–Revised (DSMQ-R), developed and validated by Schmitt et al. (17), was used to measure self-management behavior. The scale has five dimensions: eating behavior, medication taking, glucose monitoring, physical activity, and cooperation with the diabetes team. Items are rated on a 4-point Likert scale: (0 = ‘does not apply to me’ to 3 = ‘applies to me very much’). The questionnaire was administered as either a 20-item or 27-item scale (for those participants injecting insulin before meals). Scores were computed by summing the score obtained on the scale, divided by the maximum possible score (60 or 81), and multiplied by 10. The scale scores range from 0 to 10. The higher the score, the more optimal the self-management behavior. While the Cronbach’s alpha of the 20-item scale among T2DM patients in Schmitt et al. was 0.87, the Cronbach’s alpha in this study was 0.72.

##### 2.2.4.5 Financial toxicity - COST-FACIT

The Comprehensive Score for Financial Toxicity–Functional Assessment of Chronic Illness Therapy (COST-FACIT) was developed and validated by de Souza et al. (20) among cancer patients. Recently, Patel et al. validated the tool in a population with diabetes. It is an 11-item tool that comprehensively assesses the financial stress associated with the management of diabetes using a 5-point Likert scale (0 = ‘not at all’ to 4 = ‘very much’). The scale has been validated by Patel et al. (21) in a population with diabetes; it has two dimensions that measure the general financial situation (α: 0.86) and the impact of illness on financial situation (α: 0.73). Scores are computed by summing the individual item scores (reverse coding seven items), multiplying by the total items on the scale, and dividing by the number of items answered (score range: 0 – 44). In addition, a twelfth item (‘my illness has been a financial hardship to my family and me’) was added in version 2, which is not scored but is considered a summary item. The higher the score, the better the financial wellbeing. In this study, the Cronbach’s alpha values for the dimensions of general financial situation and impact of illness on financial situation were 0.73 and 0.68, respectively.

##### 2.2.4.6 Cost-related non-adherence

The cost related non-adherence measure is a 5-item questionnaire developed by Madden et al. (25) for the Medicare Current Beneficiary Survey (MCBS). The questions assess behaviors such as not filling a prescription or delaying a fill because of cost, skipping a dose or taking a smaller dose than prescribed to make medication last longer, and not obtaining prescribed medication because of cost. The items are on a 3-point Likert scale: 1 = ‘often’, 2 = ‘sometimes’, and 3 = ‘never’. Responses were dichotomized into yes (‘often’ and ‘sometimes’) and no (‘never’). For reporting purposes, ‘often’ and ‘sometimes’ were considered positive (yes), whereas ‘never’ was considered negative (no).

### 2.2.5 Quantitative data analysis

The data was analyzed using JMP Pro 18.2.1 (SAS Institute Inc., Cary, NC, USA, 1989-2025). Descriptive statistics were used to present the sample characteristics, mean scores of diabetes-related distress, self-management practices, and HbA1c. In addition, the chi-square or Fisher’s exact test was used to analyze categorical variables; whereas the independent sample t-test, Wilcoxon rank-sum test, and Kruskal-Wallis test were used to analyze continuous variables. In the regression analysis, all relevant clinical, sociodemograhic, and primary study variables were modeled using simple logistic regression. Furthermore, multivariable logistic regression models were performed, adjusting for relevant sociodemographic and clinical factors, to analyze the independent association of diabetes-related distress, diabetes self-management behavior, financial toxicity, and cost-related non-adherence with poor glycemic control. For each model, one of the factors was modeled as the primary independent variable, while covariates included age, sex, education, income, diastolic and systolic blood pressure, number of prescribed diabetic drugs, use of insulin, and study center. The significance level was considered at P <0.05.

### 2.3 Qualitative data collection and analysis

#### 2.3.1 Sample and setting

In order to explore and better understand how psychosocial, behavioral, and financial factors are associated with glycemic control, semi-structured individual interviews were conducted. The participants were purposefully selected based on the combination of glycemic control (good: < 7%; poor: ≥ 7%) and their scores on diabetes-related distress, self-management, and financial toxicity. Based on the result of the quantitative study, the median split was used to categorize the patients as follows: distressed (≥2) and non-distressed (<2); good (>7) and poor self-management behavior (≤7); and better (>20) and worse financial toxicity (≤20). Hence, a total of six possible case combinations existed within both the good and poor glycemic control groups; two participants were selected for each possible variation (good glycemic control: 6 cases, 2 each = 12; poor glycemic control: 6 cases, 2 each = 12). The participants were randomly selected from the sampling frames prepared for each possible combination. The interviews were conducted in a private room at the participants’ respective hospitals. Although 24 interviews were scheduled, only 19 interviews were completed.

#### 2.3.2 Data collection

Semi-structured interviews were conducted using an interview guide developed by the research team. The open-ended questions encompassed the source and negative effects of diabetes-related distress on adequate management, adherence to self-management behaviors, and their influence on effective control, the effect of finances on adequate management, healthcare service experiences, and the role of instrumental support in effective management (**Supplementary File 1**). Although the interviews focused primarily on the reasons underlying participants’ selection into specific categories, other relevant factors in participants’ lives were also explored. For example, a participant may have been selected because of ‘poor glycemic control’ and ‘no diabetes-related distress’; however, the same participant could have had another underlying factor (e.g., poor self-management behavior). After establishing the primary focus of the discussion in the interview, these underlying factors were further explored. This approach enabled assessment of the participants as a whole. The interview sessions were recorded and transcribed verbatim for analysis.

#### 2.3.3 Qualitative data analysis

This study used thematic analysis described by Braun and Clarke (30). The interview transcripts were managed and organized using Dedoose software (31). The analysis for the good and poor glycemic control groups was conducted independently. After the transcription, we ensured familiarization with the data and documented initial ideas. Using a theory and data-driven approach, using *in-vivo* coding, the initial codes were generated from the interviews. Afterwards, similar codes were sorted and merged into potential themes. The themes were reviewed, and internal homogeneity and external heterogeneity considered. For example, in the good glycemic control group, the themes ‘adherence to medical advice with prioritization of medication’ and ‘adherence to lifestyle changes’ were initially treated as separate themes but were later merged due to lack of external heterogeneity. As part of the refinement and naming of themes, those themes of ‘non-adherence to management due to finances’ were initially coded under diabetes self-management but later grouped under financial toxicity. The themes were defined and named based on their representation of the factors and data within the interviews.

### 2.4 Data integration

Since the quantitative strand of the study informed the qualitative strand, the result of the qualitative analysis was used to explain the quantitative findings. This provided deeper understanding and insight into the findings of the quantitative strand. This finding was presented using a joint display, and findings were integrated at the level of discussion.

### 2.5 Ethical consideration

This study was conducted in accordance with the Declaration of Helsinki, the Ethical Guidelines on Clinical Studies of the Ministry of Health, Labor and Welfare of Japan, and the Nigerian National Code for Health Research Ethics. It was approved by the Ethical Committee for Epidemiology of Hiroshima University (E2024-0081) and the CMUL Health Research Ethics Committee (CMUL/HREC/09/24/1629), Nigeria. The participants were provided with sufficient information and written informed consent was obtained.

## 3 Results

### 3.1 Glycemic control

Out of the 378 participants recruited, a total of 355 participants with complete responses and valid HbA1c results (response rate: 93.9%) were included in the final analysis. The mean HbA1c was 7.04% (SD: 2.2). Based on the predefined classification used in this study, 43% (n= 152) of respondents had poor glycemic control (HbA1c ≥7%) (**Table 1**).

**Table 1:** Glycemic control among patients attending the outpatient clinic.

Variable	Full sample (N = 355), n (%)
HbA1c (%), mean (SD)	7.04 (2.2)
Good (<7)	203 (57.2)
Poor (≥7)	152 (42.8)

### 3.2 Demographic and clinical characteristics

The mean age of the participants was 61.5 years of age, and approximately 40% were within the age range of 55-64 years. The majority of participants were female (77%, n = 272), of Yoruba ethnicity (85%, n = 302), married (63%, n = 225), had completed secondary education or above (66%, n = 233), and were employed (60%, n = 213). In total, 40% (n = 133) earned less than 60,000 naira, and 87% (n = 302) had no medical insurance. In addition, 93% (n = 330) did not consume alcohol, all participants were non-smokers (100%, n = 354), and 57% (n = 204) and 42% (n = 149) identified as Christians and Muslims, respectively. The mean duration of T2DM was 9.6 years (SD: 7.7). Participants were prescribed an average of 2 diabetic medications (SD: 0.8), while the total number of drugs was 5.6 (SD: 2). Systole was 138.4 mmHg (SD: 21.1), and diastole was 81.0 mmHg (SD: 12.4). The mean BMI was 28.8 Kg/m² (SD: 6.2), and 72% (n = 246) were either overweight or obese. Approximately 29% and 71% were receiving monotherapy and polytherapy (≥2), respectively. The majority (82%, n = 292) were on oral drugs only, and 18% were prescribed insulin either alone or in combination (**Table 2**).

**Table 2:** Demographic and clinical differences according to glycemic control among patients attending the outpatient clinic.

Variables	Glycemic control		P-value^a^	Full sample (N = 355), n (%)
	Good (HbA1c <7%)	Poor (HbA1c ≥7%)		
Age, mean (SD)	63.3 (9.7)	59.2 (9.6)	<0.001	61.5 (9.8)
<55	32 (38.6)	51 (61.4)	<0.001	83 (23.4)
55 - 64	80 (57.6)	59 (42.4)		139 (39.1)
≥65	91 (68.4)	42 (31.6)		133 (37.5)
Sex				
Female	157 (57.7)	115 (42.3)	0.711	272 (76.6)
Male	46 (55.4)	37 (44.6)		83 (23.4)
Ethnicity				
Yoruba	177 (58.6)	125 (41.4)	0.517	302 (85.1)
Igbo	10 (47.6)	11 (52.4)		21 (5.9)
Edo	6 (60.0)	4 (40.0)		10 (2.8)
Others	10 (45.4)	12 (54.6)		22 (6.2)
Religion				
Islam	86 (57.7)	63 (42.3)	0.614	149 (42.0)
Christianity	115 (56.3)	89 (43.7)		204 (57.4)
Others	2 (100.0)	0 (0.0)		2 (0.6)
Marital status				
Single	3 (60.0)	2 (40.0)	0.663	5 (1.4)
Married	124 (55.1)	101 (44.9)		225 (63.4)
Divorced	11 (68.8)	5 (31.2)		16 (4.5)
Widowed	65 (59.6)	44 (40.4)		109 (30.7)
Education				
No formal education	15 (57.7)	11 (42.3)	0.479	26 (7.3)
Primary	56 (58.3)	40 (41.7)		96 (27.0)
Secondary	61 (51.7)	57 (48.3)		118 (33.3)
Tertiary	71 (61.7)	44 (38.3)		115 (32.4)
Employment				
Unemployed	27 (52.9)	24 (47.1)	0.012	51 (14.5)
Employed	113 (53.0)	100 (47.0)		213 (60.3)
Retired	63 (70.8)	26 (29.2)		89 (25.2)
Monthly income				
No income	65 (60.2)	43 (39.8)	0.814	108 (32.3)
<₦60,000 (<$40)	74 (55.6)	59 (44.4)		133 (39.8)
₦60,000 - 119,999 ($40 - 79)	23 (52.3)	21 (47.7)		44 (13.2)
≥₦120 (≥$80)	28 (57.1)	21 (42.9)		49 (14.7)
Smoking				
Yes	0 (0.00)	1 (100.0)	0.428	1 (0.3)
No	203 (57.3)	151 (42.7)		354 (99.7)
Alcohol				
Yes	13 (52.00)	12 (48.0)	0.588	25 (7.0)
No	190 (57.6)	140 (42.4)		330 (93.0)
Duration of DM, mean (SD)	9.6 (8.1)	9.5 (7.1)	0.89	9.6 (7.7)
<5	81 (58.7)	57 (41.3)	0.58	138 (40.1)
≥5	114 (55.3)	92 (44.7)		206 (59.9)
Type of DM drug, mean (SD)				
Oral	183 (62.7)	109 (37.3)	<0.001	292 (82.2)
Oral and Insulin	19 (31.1)	42 (68.9)		61 (17.2)
Insulin	1 (50.00)	1 (50.00)		2 (0.6)
Insulin use				
Yes	20 (31.8)	43 (68.2)	<0.001	63 (17.8)
No	183 (62.7)	109 (37.3)		292 (82.2)
Number of DM drugs, mean (SD)	1.8 (0.7)	2.2 (0.8)	<0.001	2.0 (0.8)
1	73 (73.0)	27 (27.0)	<0.001	100 (28.8)
2	95 (55.6)	79 (44.4)		171 (49.1)
≥3	30 (39.0)	47 (61.0)		77 (22.1)
Total number of drugs, mean (SD)	5.5 (2.1)	5.7 (2.0)	0.324	5.6 (2.0)
Comorbidity				
Yes	196 (58.51)	139 (41.49)	0.193	335 (90.30)
No	17 (47.22)	19 (52.78)		36 (9.70)
Insurance				
Yes	25 (53.3)	21 (45.7)	0.646	46 (13.2)
No	175 (58.0)	127 (42.0)		302 (86.8)
Insurance type				
NHIS	11 (68.8)	5 (31.2)	0.452	16 (36.4)
Ilera Eko	12 (48.0)	13 (52.0)		25 (56.8)
Others	2 (66.7)	1 (33.3)		3 (6.8)
Blood pressure (mm Hg)				
Systolic	137.5 (20.5)	139.6 (21.9)	0.361	138.4 (21.1)
Diastolic	79.0 (11.8)	83.7 (12.7)	<0.001	81.0 (12.4)
BMI (kg/m²), mean (SD)	29.1 (6.3)	28.3 (6.0)	0.188	28.8 (6.2)
Underweight (<18.5)	1 (20.0)	4 (80.00)	0.244	5 (1.5)
Normal (18.5 - 24.9)	51 (56.0)	40 (44.0)		91 (26.6)
Overweight (25 -29.9)	72 (56.2)	56 (43.8)		128 (37.4)
Obese (≥30)	74 (62.7)	44 (37.3)		118 (34.5)

a. P-values were generated using the t-test, Wilcoxon rank-sum test, chi-square test, or Fisher’s exact test.

DM, diabetes mellitus; SD, standard deviation; BMI, body mass index; mm Hg, millimeters of mercury.

**Table 2** shows differences in glycemic control according to respondents’ profiles. Glycemic control status was dependent on age, employment status, diastolic blood pressure, use of insulin, and number of antidiabetic medications. The mean ages of those with good and poor glycemic control were 63.3 and 59.2 years, respectively. Differences in glycemic control across age groups were also evident (P <0.001), with more likelihood of poor control among those <55 years (61.4%). There was significant association between glycemic control and employment status. Retired participants were more likely to have good glycemic control; the percentages of poor glycemic control were 47%, 47%, and 29% among unemployed, employed, and retired participants, respectively (P = 0.012). The type of medication showed a significant association, as participants using only oral drugs tended to have good glycemic control than those using oral and insulin or insulin alone (P <0.001). Those who were not prescribed insulin had a higher prevalence of good glycemic control (62.7%) compared with those prescribed insulin (either alone or in combination: 31.8%). Moreover, glycemic control was associated with the number of antidiabetic medications used. The percentages of good glycemic control were 73%, 55.6%, and 39% among participants using one, two, and three or more antidiabetic medications, respectively.

### 3.3 Diabetes self-management, diabetes-related distress, financial toxicity, and cost-related non-adherence

The mean diabetes self-management score was 6.8 (SD:1.2). Differences in self-management practices according to glycemic control status were observed, with significantly lower self-management scores among participants with poor glycemic control (6.6; SD: 1.2) than among those with good glycemic control (7.0; SD: 1.2). Among the dimensions of self-management, a higher score for eating behavior was associated with good glycemic control (good: 6.6 vs. poor: 5.9; P = 0.002). However, there were no significant associations between glycemic control and scores for medication taking, glucose monitoring, physical activity, and cooperation with the diabetes team. The median self-management behavior score was 7. Using a median split to categorize the respondents into good and poor self-management practices (>7 vs ≤ 7), 56% (n = 198) of participants were classifies as having poor self-management behavior (**Table 3**).

**Table 3:** Diabetes self-management, diabetes-related distress, financial toxicity, and cost-related nonadherence difference in glycemic control.

Variables	Glycemic control		P-value^a^	Full sample (N = 355)
	Good HbA1c (<7%)	Poor HbA1c (≥7%)		
DSM, mean (SD)	7.0 (1.2)	6.6 (1.2)	0.002	6.8 (1.2)
Eating Behaviour	6.6 (2.0)	5.9 (2.1)	0.002	6.3 (2.1)
Medication Taking	8.6 (2.2)	8.5 (2.3)	0.925	8.6 (2.2)
Glucose Monitoring	7.0 (3.0)	6.6 (3.1)	0.16	6.8 (3.0)
Physical Activity	7.0 (2.7)	6.9 (2.7)	0.753	6.9 (2.7)
Diabetes Team	7.6 (1.2)	7.4 (1.5)	0.089	7.5 (1.4)
DRD, (mean SD)	1.7 (0.7)	1.9 (0.7)	<0.001	1.8 (0.7)
Emotional Burden (EB)	2.3 (1.1)	2.7 (1.2)	0.002	2.4 (1.2)
Regimen Distress (RD)	1.7 (0.8)	2.0 (0.9)	<0.001	1.8 (0.9)
Physician Distress (PD)	1.3 (0.6)	1.3 (0.6)	0.109	1.3 (0.6)
Interpersonal Distress (ID)	1.4 (0.9)	1.4 (1.0)	0.954	1.4 (0.9)
Financial Toxicity (FT), mean (SD)	20.1 (9.1)	19.5 (8.9)	0.419	19.8 (9.0)
General Financial Situation	12.9 (6.1)	12.9 (5.8)	0.754	12.9 (6.0)
Impact on Financial Situation	7.2 (3.9)	6.6 (4.1)	0.164	6.9 (4.0)
Cost-Related Nonadherence (CRN)^b^				
Yes	89 (52.1)	82 (47.9)	0.065	171 (48.3)
No	113 (61.8)	70 (38.2)		183 (51.7)
	n (%)	n (%)	P-value^a^	Full sample (N = 355), n (%)
DSM				
Good (>7)	99 (63.1)	58 (36.9)	0.046	157 (44.2)
Poor (≤7)	104 (52.5)	94 (47.5)		198 (55.8)
Financial toxicity				
Better (>20)	101 (61.6)	63 (38.4)	0.11	164 (46.3)
Worse (≤20)	101 (53.2)	89 (46.8)		190 (53.7)
DRD				
Yes (≥2)	50 (43.9)	64 (56.1)	<0.001	114 (32.1)
No (<2)	153 (63.5)	88 (36.5)		241 (67.9)

a. P-value generated by Wilcoxon/Chi-square

b. CRN: Defined as practicing at least one of the cost-coping measures in the past 6 months: Decided not to buy medication, Skip medication, Took small dose, Delayed buying medication, Did not buy medication

DSM, Diabetes self-management; DRD, Diabetes-related distress

The mean diabetes-related distress score was 1.8 (SD: 0.7). There was a significant difference in the mean score of diabetes-related distress between participants with good and poor glycemic control, with higher scores was associated with poor glycemic control (P <0.001). Among the dimensions of diabetes-related distress, higher scores for emotional distress and regimen distress were associated with poor glycemic control. However, there were no associations between glycemic control status and physician distress or interpersonal distress scores. Based on the established classification, 32% of respondents reported diabetes-related distress (**Table 3**).

The mean financial toxicity (FT) was 19.8 (SD: 9). Although participants with poor glycemic control perceived greater financial stress (low score), this difference was not significantly associated with glycemic control (P = 0.419). This was also true for the dimensions of general financial situation and impact of illness on financial situation. Based on the median split of 20 (Better: > 20; ≤ worse: 20), approximately 54% of respondents reported financial stress (**Table 3**).

In terms of cost-related non-adherence, almost half (48.3%; n = 171) of the participants reported practicing at least one of the maladaptive cost-coping strategies. However, cost-related non-adherence was not associated with glycemic control (P = 0.065).

### 3.4 Financial toxicity and cost-related non-adherence

**Figure 1:**
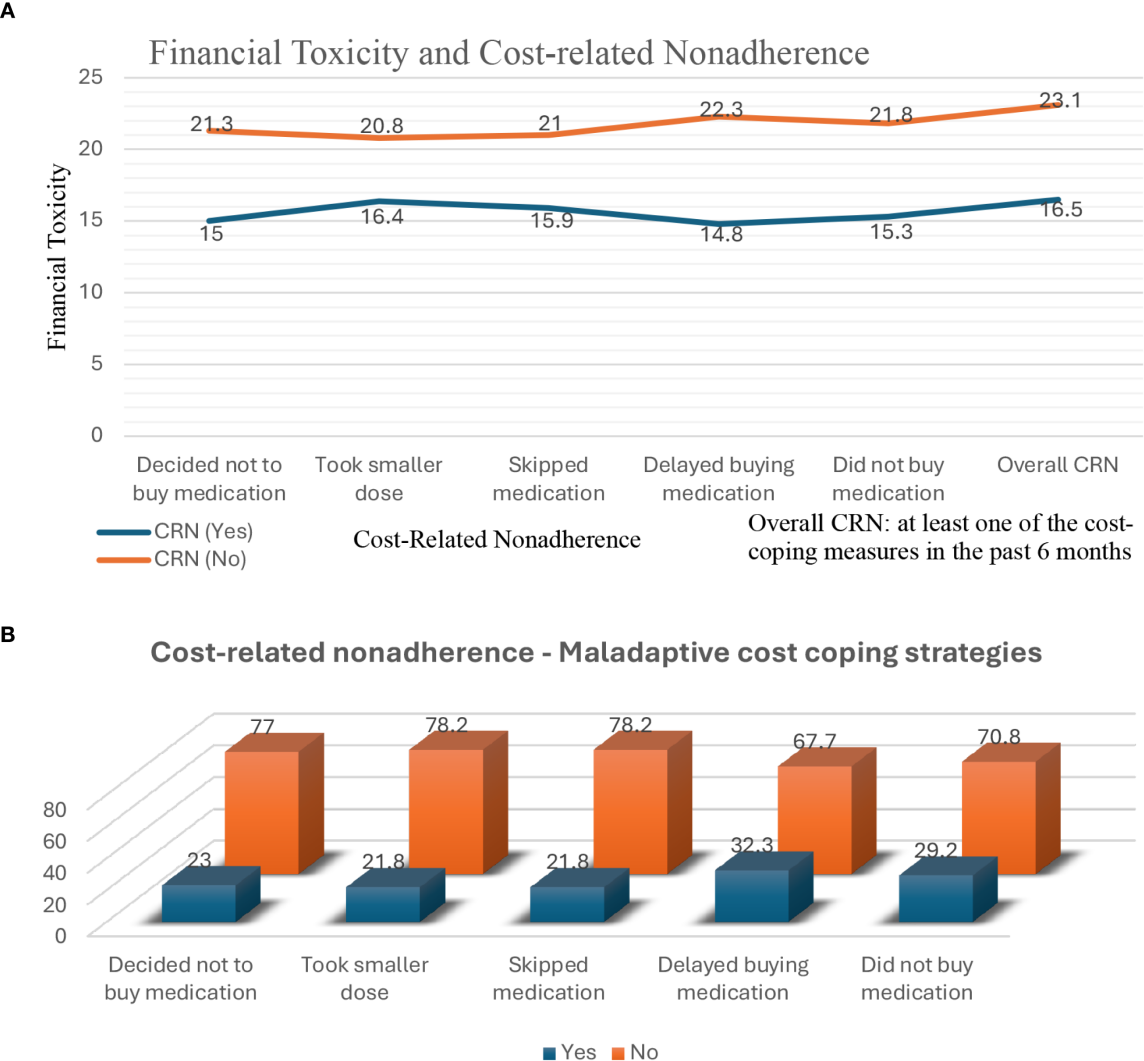
**(a)** Association between financial toxicity and cost-related non-adherence. **(b)** Cost-related non-adherence.

Financial toxicity was associated with cost-related non-adherence, as greater financial stress was reported among participants who engaged in the maladaptive cost-coping strategies (**Figure 1a**). This association was consistent across all cost-coping strategies (P<0.001). Among the cost-coping strategies, delaying the purchase of medications was the most common (32.3%; n = 113), followed by not purchasing medications because of cost (29.2%; n =103) (**Figure 1b**).

### 3.5 Determinants of poor glycemic control

**Table 4** presents the regression models for factors associated with poor glycemic control. In the simple logistic regression model, age younger than 55 years (OR: 3.45; 95% CI: 1.96 - 6.18), use of two (OR: 2.16; 95% CI: 1.28 - 3.73) or three or more antidiabetic medications (OR: 4.24; 95% CI: 2.26 - 8.10), insulin use (OR: 3.61; 95% CI: 2.04 - 6.57), diastolic blood pressure (OR: 1.03; 95% CI: 1.01 - 1.05), poor diabetes self-management behavior (OR: 1.54; 95% CI: 1.01 - 2.37), diabetes-related distress (OR: 2.23; 95% CI: 1.42 - 3.52), and study site B (OR: 1.92; 95% CI: 1.16 - 3.22) were significantly associated with poor glycemic control.

**Table 4:** Socioeconomic, clinical, behavioral, psychosocial, and finacial factors associated with poor glycemic.

Variables	Model 1	Model 2	Model 3	Model 4	Model 5
	COR (95% CI)	AOR (95% CI)	AOR (95% CI)	AOR (95% CI)	AOR (95% CI)
Age category					
<55	**3.45 (1.96 - 6.18)**	**2.88 (1.33 - 6.20)**	**2.69 (1.24 - 5.84)**	**3.30 (1.54 - 7.10)**	**3.22 (1.50 - 6.90)**
55 - 64	1.60 (0.98 - 2.64)	1.28 (0.69 - 2.39)	1.32 (0.71 - 2.46)	1.30 (0.69 - 2.46)	1.36 (0.74 - 2.51)
≥65	Ref (1.00)	Ref (1.00)	Ref (1.00)	Ref (1.00)	Ref (1.00)
Sex					
Female	0.91 (0.56 - 1.50)	0.72 (0.38 - 1.37)	0.78 (0.41 - 1.48)	0.76 (0.41 - 1.44)	0.78 (0.41 - 1.47)
Male	Ref (1.00)	Ref (1.00)	Ref (1.00)	Ref (1.00)	Ref (1.00)
Income					
No income	0.88 (0.45 - 1.76)	0.76 (0.31 - 1.85)	0.09 (0.37 - 2.18)	0.86 (0.36 - 2.09)	0.84 (0.35 - 2.04)
<₦60,000 (<$40)	1.06 (0.55 - 2.08)	1.07 (0.46 - 2.46)	1.26 (0.55 - 2.88)	1.19 (0.52 - 2.75)	1.44 (0.50 - 2.62)
₦60,000-119,999 ($40-79)	1.22 (0.54 - 3.78)	1.90 (0.71 - 5.07)	1.90 (0.71 - 5.04)	1.90 (0.72 - 5.04)	1.99 (0.74 - 5.37)
≥₦120 (≥$80)	Ref (1.00)	Ref (1.00)	Ref (1.00)	Ref (1.00)	Ref (1.00)
Education					
None	Ref (1.00)	Ref (1.00)	Ref (1.00)	Ref (1.00)	Ref (1.00)
Primary	0.97 (0.41 - 2.39)	0.60 (0.20 - 1.84)	0.61 (0.20 - 1.88)	0.61 (0.20 - 1.85)	0.59 (0.19 - 1.82)
Secondary	1.27 (0.54 - 3.07)	0.69 (0.22 - 2.09)	0.76 (0.25 - 2.31)	0.75 (0.25 - 2.25)	0.74 (0.24 - 2.22)
Tertiary	0.85 (0.36 - 2.04)	0.32 (0.10 - 1.06)	0.35 (0.11 - 1.15)	0.34 (0.11 - 1.09)	0.34 (0.11 - 1.10)
Number of Diabetic drug					
1	Ref (1.00)	Ref (1.00)	Ref (1.00)	Ref (1.00)	Ref (1.00)
2	**2.16 (1.28 - 3.73)**	**2.31 (1.23 - 4.34)**	**2.10 (1.12 - 3.95)**	**2.17 (1.16 - 4.06)**	**2.13 (1.14 - 4.00)**
≥3	**4.24 (2.26 - 8.10)**	**4.07 (1.85 - 8.92)**	**4.03 (1.83 - 8.86)**	**4.09 (1.88 - 8.90)**	**4.23 (1.93 - 9.30)**
Insulin					
Yes	**3.61 (2.04 - 6.57)**	**3.34 (1.54 - 7.22)**	**3.15 (1.47 - 6.77)**	**3.17 (1.49 - 6.76)**	**3.17 (1.48 - 6.81)**
No	Ref (1.00)	Ref (1.00)	Ref (1.00)	Ref (1.00)	Ref (1.00)
Blood Pressure					
Systole	1.00 (0.99 - 1.01)	0.99 (0.98 - 1.01)	0.99 (0.98 - 1.01)	0.99 (0.98 - 1.01)	0.99 (0.98 - 1.01)
Diastole	**1.03 (1.01 - 1.05)**	1.02 (0.99 - 1.05)	1.03 (0.99 - 1.06)	1.03 (0.99 - 1.05)	1.02 (0.99 - 1.05)
Study Site					
Site A	1.27 (0.73 - 2.22)	1.64 (0.81 - 3.35)	1.50 (0.74 - 3.05)	1.51 (0.74 - 3.06)	1.57 (0.78 - 3.21)
Site B	**1.92 (1.16 - 3.22)**	**3.39 (1.71 - 6.74)**	**2.88 (1.49 - 5.57)**	**2.79 (1.45 - 5.37)**	**2.98 (1.54 - 5.81)**
Site C	Ref (1.00)	Ref (1.00)	Ref (1.00)	Ref (1.00)	Ref (1.00)
DSM					
Good	Ref (1.00)	Ref (1.00)	-	-	-
Poor	**1.54 (1.01 - 2.37)**	**2.02 (1.18 - 3.45)**	-	-	-
DRD					
Yes	**2.23 ( 1.42 - 3.52)**	-	**2.13 (1.22 - 3.72)**	-	-
No	Ref (1.00)	-	Ref (1.00)	-	-
Financial Toxicity	0.99 (0.97 - 1.02)		-	1.01 (0.98 - 1.04)	-
CRN					
Yes	1.49 (0.98 - 2.27)	-	-	-	1.31 (0.78 - 2.19)
No	Ref (1.00)	-	-	-	Ref (1.00)

DSM, Diabetes self-management; DRD, Diabetes-related distress; CRN, Cost-related nonadherence

Bolded values indicate P < .05

After adjustment for sociodemographic and clinical factors, only diabetes-related distress and diabetes self-management were significant in their models. Financial toxicity and cost-related non-adherence were not determinants of poor glycemic control. Participants with poor self-management behavior were 2.02 times more likely to have poor glycemic control, whereas participants with diabetes-related distress had 2.23 times higher odds of poor glycemic control. Although diastolic blood pressure was significantly associated with poor glycemic control in the unadjusted model, this effect was attenuated and non-significant in the adjusted models. However, the covariates of age, number of diabetic drugs, insulin, and study site B were significantly associated with poor glycemic control in all the adjusted models.

### 3.6 Qualitative findings

**Table 5** presents the integration of quantitative and qualitative findings using joint display. The themes are organized according to diabetes self-management, diabetes-related distress, and financial toxicity. An additional miscellaneous theme was created and addresses healthcare service delivery. Themes were identified across both good and poor glycemic control groups. For the groups of poor glycemic control: diabetes self-management: (1) ‘Difficulty adapting to lifestyle changes’, (2) ‘Medication non-adherence due to fear of aftereffect from prolonged use or polypharmacy,’ and (3) ‘Supportive therapy with traditional prescription’; Diabetes-related distress: (1) ‘Acceptance of disease as a challenge’ and (2) ‘Temporal distress from financial constraints’; Financial toxicity: (1) ‘Expensive, but managing with financial support’ and (2) ‘Non-adherence to treatment management due to finances’. An instrumental support theme common to both good and poor glycemic control groups was ‘re-envisioning healthcare service delivery’.

**Table 5:** Joint display of quantitative and qualitative findings.

Quantitative variables	Qualitative interview	
	Good Glycemic Control	Poor Glycemic Control
**Diabetes-related distress (DRD)**	**Theme: Acceptance of disease as a challenge** Describes the acceptance of disease as part of life. “And I believe any human being have one sickness or the other.” (Interview 2) “Yes, it’s a problem. I don’t neglect the diabetes because I know that if I don’t keep myself. I know the sugar will rise and if it rises, it will affect me. ” (Interview 15) **Theme: Distress in management and complication experience** Describes patients feeling of distress from the lifelong of diet control and experience of diabetes-related complication. “Actually, it causes a bit of distress. reasons are some people will see me for the first time looking. My leg is not smelling; I manage it, perfectly. And I even thank god, the extent is reducing." (Interview 5) “Whenever I want to buy something like amala, I would ask: “I hope white flour is not included”. Because if anything happens to me, you will be responsible. The distress or problem is much. I could not eat fufu or lafun. It is distressful." (Interview 12)	**Theme: Acceptance of disease as a challenge** Describes the acceptance of disease as part of life. “The regimen is not a real problem because it is now a challenge; I just have to face it. God is there to make it better.” (Interview 4) “I don't think it really affects me in any of those. I have seen it as somethng manageable. You understand. It is in my family history; I have seen my parents doing fine irrespective of the disease. I don't have much stress about the effect.” (Interview 14) **Theme: Temporal distress from financial constraint** Describes patients temporary feeling of distress from the inability to buy medication due to financial constraint. “It affects because when I do not have enough money to buy the drugs. Or the money is not nough for what has been prescribed and there is no other means. Maybe there is no money from my business; It affects. But whenever I am able to buy, the problem is gone.” (Interview 4)
**Diabetes self-management (DSM)**	**Theme: Adherence to lifestyle changes with prioritization of medication** Describes the adherence to the instructions provided during visits, lifestyle modification through knowledge of self-management, previous experience of complication, and realization of medication adherance as priority. “It is using the drugs as prescribed. They said we should be using our drugs. That is what I think is important, and regular clinic visit.” (Interview 9) “Pension. So that one, I have already made it for buying medicine. I don't use it for any other thing. is to keep my life. You understand. Not everything for food.” (Interview 2) “Whenever we visit the clinic, they tell us what to eat and what not. Before, they used to advise that we should eat some certain foods like beans, vegetables, fruits..... After they did more findings, they told us we can eat anything, but it shouldn’t be much. For example, if it is swallow food, it should not be more than once in a day.” (Interview 19) "I have abandoned rice for a long; it is only in a while. I do not eat jollof rice. Even when they serve me rice and I tell them it is enough; they would question the portion as “when it is not for a kid”. I would just tell them: “let me finish this even if I have to eat more”. As for water, when I fill this stomach with it, everything is alright. “(Interview 11) “Even if I don’t have money, I borrow from the bank applications. I do not want the drug to affect the management because I have had a bad experience with diabetes.” (Interview 10). **Theme: Supportive therapy with traditional prescription** Describes adoption of alternative medicine or practices “So I put it inside coconut water. I drink it early in the morning, before I eat anything. And I used to drink guava. I cook and drink it. Yeah. So I use herb by myself; I drink it. That’s why. I don’t eat anyhow food. I don't eat bread. If I want to eat bread, I eat wheat bread. If I want to drink pap, I don't put sugar. I don't use sugar at all.” (Interview 15) “How I was able to manage it to low level is benecid (sesame seed); however; they would instill fear in me that it could affect my liver and kidney. You could take a video. Whenever I eat my food with sesame seed and water, my body becomes ok. I frequently eat it. I do not want to say much.” (Interview 12)	**Theme: Difficulty adapting to lifestyle changes** Describes patients experience of being aware of the the need for lifestyle change but experiencing difficulty in implementation due to the need to eat to satisfaction, unawareness of complication, and inability to give up previous habit. “As human being, we do not easily accept to give up what we have been eating. It is only when we realise it is dangerous” (Interview 4). “That may not be too strictly possible. I have not been doing that, possibly. Too strictly. So definitely I can't say I am 100% or too good at my eating habits. They said we should not eat in between meals. I eat in between meals. So my eating habits has not been good. My eating is very poor. Even at the clinic we complain that it's not possible for me to just eat a fist ooo. And the nurses would say, all of us, majority of use, we will achieve satiety. We will achieve satiety or not, it is an individual problem” (Interview 1) “I said that it is unhealhty food. There is nothing than that. Because now, when I got here, I went down there and ate rice. Then I buy chips to eat it because I know that rice alone is not satisfying. So if I don’t have something to eat with it, I won't be satisfied. So I bought the dried plantain to eat it. It is still part of unhealthy eating. If its before, I would have gotten soda. So now that it is affecting my eyes, I get water. If God would assist me to stop the unhealthy eating, I think I will be ok. It is affecting my eyes now.” (Interview 17) "Whenever I want to eat, I want to eat to my satisfaction. If it's a bowl of rice that will satisfy me at that point in time, I will take it. Which diabetes patient should not do. As at now, I am trying. I'm trying to adjust." (Interview 8) **Theme: Medication nonadherence due to fear of aftereffect from prolonged use or polypharmacy** Describes patient adjustement of medical treatment due to self-believe and misconception of kidney or liver damage from prolonged use of medication. “Or at times, I normally fear kidney or liver issue. Like now, the doctor prescribed 2 types of drugs, the other doctor prescribed other drugs, making like 6 or 10 types of drugs. And too much of drugs normally damage. I will now be like, I can sit for one week without using medicine because I don’t want a situation that I start using them and later damage another thing in my body.” (Odan 17) “I am on insulin, as at yesterday, the doctor said I should be using 16. Even he pushed to about 18. I started from 14, 16 and yesterday he said I should be using 18. This yesterday night I didn't use 18, I used 16. Because I asked him yesterday that what is the after effect if I use 18, he said there is no side effect, but I cannot believe him. So yesternight, I just gave myself the injection of 16. I used 16 yesterday, and I woke up well.” (Interview 1) **Theme: Supportive therapy with traditional prescription** Describes adoption of alternative medicine or practices “And my wife, she's equally trying. She will introduce herbal concotion. Daddy, orthodox medicine is not enough; you should also use this. We combine everything.” (Interview 8) “I am usually afraid to use traditional medicine. The only one i use is coconut water and agbebi from a friend.” (Interview 6)
**Financial toxicity**	**Theme: Expensive, but managing with financial support** Describes patient experience of financial stress with support from children and family members “Even my income is nothing to write home about. It's just the grace of God and some support. It is not what we earn that we are spending.” (Interview 5) "It is huge expenses. Before, when I started, I did not really spend much; However, If I take 60,000 to 70,000 to the drug store. I would buy everything together with the hypertensive drugs. It is taking a lot of money from me." “It is the children. They send the money for drug every month; they would for food the next month. The drugs are for every month; some are not even up to a month.” (Interview 11) “I also have a sister and my child who helps. I have not been able to work since the sickness; I only stay at home. Especially during this fasting period, they are supporting me.” (Interview 10) **Theme: Diet control to reduce money spent on drugs** Describes patients strategy to to judiciously implement diet control in order to reduce the financial stress of purchasing drugs. “That is why I controlled my food because I know that I don't have money to control my diabetes.”(Interview 15) “it affects in a particular place, and we manage in the other way. In other way in the sense that we're trying to watch, you understand, the intake of our food, especially my own food.” (Interview 2)	**Theme: Expensive, but managing with financial support** Describes patient experience of financial stress with support from children and family members It’s much ooo. I am not comfortable. Supposed I did not have that kind of person, that is what kills people. When you don’t have money and you are sick.” (Interview 1) “Everyone of them has or knows which to buy. They do not even default. When the doctor said the diabetes cannot go away, that I have to manage it for life. The children did not relent or become fedup. They already know what to buy at the end of the month. I do not spend any penny.” (Interview 3) “It is from the business capital, if there is no one to gift. The health is first even if I cannot restock.” (Interview 4) “I strive by spending my business capital to buy the drugs; it ends up affecting my stock that I would not be able to sell.” (Interview 18) **Theme: Nonadherence to management due to finances** Describes patient experience of cost-related nonadherence to drug and diet. “Sometimes, the doctor advises I use one in the morning and one at night. I would just think that what if it gets exhausted. How am I going to be able to purchase another. I would just use only one.” (Interview 18) “Sometimes, I manage myself. But my drugs, I use it everytime. But food sometimes, nigeria, maybe you have beans. They told us if we want to cook rice, we should cook it with beans. Whatever you want to eat, maybe fufu, eat it with much vegetables. In nigeria of today, if you want to cook small vegetable, you will spend more than 2,000 naira. Just small small vegetable to use immediately, you will spend more than 2000 naira. Its not with meat; just the vegetable will cost more than 2000 naira. Everything in nigeria, you will manage small small.” (Interview 6)
**Instrumental Support**	**Theme: Reenvisioning the healthcare service delivery** “I feel there should be more education about diabetes and diabetes management and control in this our community, because the information they give is not enough, although they still refer patients to dietitians. But of course, sometimes because of crowd.. or they assume that people. So they don't really give that solid information. It could be better. That's what I'm trying to say.” (Interview 16) **Theme: Cost of drug reduction** “We also need the reduction of drugs. The drugs here is cheaper than outside; however, they would need the doctors prescription before selling.” (Interview 10) “Yes, it is very important. I cant tell them to give me money for food, but for health, they should reduce the cost so I would be able to buy it.” (Interview 15)	**Theme: Reenvisioning the healthcare service delivery** “There is nothing being taught here than drugs. It is only drugs. There is nothing. They will just say go and use your drugs. They should try and be explaining very well. It is very important. Diabetes is not a minor disease. It could kill fast. The high and low are bad. They should advise and explain deeply. They would tell us to go manage ourselves and send us to the dietitian. If we just come, the nurses will just do BP and blood sugar testing and tell us to see the Dr; that’s is all they do. When we get to the Dr, they will just ask about our drug compliance, and we tell them. The other time they go further is when the blood sugar is high; at least if the blood sugar is high, it is only a day.” (Interview 6) “When I complain to them that it is high, they would tell me to observe what I eat. I do tell them I still eat the same way. When they say that, I would just say: maybe we should not eat again. (Interview 7)

Bolded sentence = Themes.

## 4 Discussion

In this study, participants from multiple general hospitals were used to assess the levels of diabetes-related distress, self-management behavior, financial toxicity, and cost-related non-adherence, and to examine their associations with glycemic control. Diabetes-related distress and diabetes self-management were associated with glycemic control, whereas financial toxicity and cost-related non-adherence did not reach statistical significance. Moreover, the covariates of age, number of antidiabetic drugs, insulin, and study site B were significantly associated with poor glycemic control.

HbA1c is a useful biomarker of long-term glycemic control. In our study, the mean HbA1c was 7.04 (SD: 2.2). Based on the classification, nearly half (43%) of the participants had poor glycemic control. This finding is consistent with reports from other studies conducted at different hospital levels across the country. However, this percentage is lower than the 67% reported by Omotosho et al. (6) and Onwuchuluba et al. (5), but higher than the 30% reported in Ogbera and Adeyemi-Doro (32) in the same state. The range and difference in the percentage reported might be due to the different hospital level, region, and indicator of glycemic control (FBS, HbA1c).

The mean self-management behavior score was 6.8 (SD:1.2). This is similar to findings form one of the studies on the validation of the questionnaire by Schmitt et al. (17) and Babatunde and Onu (33) in Nigeria. Approximately 56% of participants reported poor self-management behavior, and consistent with previous studies (17, 34), poor self-management was associated with poor glycemic control; those with poor self-management were 2.02 times more likely to experience poor glycemic control. Adequate self-management is the cornerstone of good glycemic control; in particular, eating behavior appeared to be a significant contributing factor to this difference. This highlights the importance of diet control, as some patients are reluctant or reported difficulty in making lifestyle changes. This is further supported by the qualitative theme of ‘difficulty adapting to lifestyle changes’, with patients revealing that eating ‘a fist’ or portion control ‘may not be too strictly possible’ and inability to adjust eating habits, such as “late night eating”, urge to “eat to satisfaction”, and lack of self-control. The system is largely dependent on doctor-patient instruction and limited involvement of dietitians and other allied health professionals in the management of diabetes patients. Iregbu et al. (35) found that healthcare professionals readily expect compliance to instructions as they know what is best for the patient; moreover, dietitians enforce strict dietary plans that patients have expressed as “is what you are asking me to do possible” and some refused outright. Although the bulk of responsibility of self-management lies with the patient, sound education, motivational interviewing, and streamlined support should be integrated into the system. This could help struggling patients to understand the need for lifestyle changes and support modifications. While the quantitative study did not reveal significant difference in medication use, qualitative findings suggested that participants with poor glycemic control might not have been adhering to medications. Notably, the theme “Medication non-adherence due to fear of aftereffect from prolonged use or polypharmacy” found that some participants either “self-adjust” the medication or “stay for a week” without using medication due to fear of aftereffects of polypharmacy. There was misconception that long-term usage of prescribed medications could lead to kidney damage. A patient stated: “Or at times, I normally fear kidney or liver issue. Like now, the doctor prescribed 2 types of drugs, the other doctor prescribed other drugs, making like 6 or 10 types of drugs. And too much of drugs normally damage. I will now be like, I can sit for one week without using medicine because I don’t want a situation that I start using them and later damage another thing in my body.” [sic].

Approximately one-third (32%) of the participants had diabetes-related distress worthy of clinical attention. This is lower than the 45.1% (6) and 55.1% (5) reported in studies conducted in the same locality. There is possibility that the higher good glycemic control in this study reflects the low occurrence of distress. There has been varying findings on diabetes-related distress, with prevalence of 31% in Brazil (36), 36.8% in Ethiopia (37), and 44.7% in Ghana (38). In this study, participants who reported diabetes-related distress had 2.13 times higher odds of poor glycemic control than those without distress. This finding is consistent with Dalsgaard et al. (39), who reported 1.8 times higher odds of poor glycemic control among individuals with distress. Contrary to previous studies on the indirect relationship between diabetes-related distress and glycemic control (40, 41), the qualitative theme of “acceptance of disease as a challenge” revealed that those with diabetes-related distress did not relent or lose motivation in continuing disease management. A participant with distress stated: “The regimen is not a real problem because it is now a challenge; I just have to face it. God is there to make it better.” This supports the finding of the *post-hoc* analysis, which found that self-management does not mediate the effect of diabetes-related distress on glycemic control. Notably, two themes emerged as different sources of distress beyond the dimensions captured by the diabetes-related distress scale. The themes ‘finances potentiate distress’ and ‘distress associated with management and complication’ highlight the need to expand the understanding of diabetes-related distress; these sources are similar to healthcare access and stigma, which have been identified in the recent modification of the scale (42).

In total, 64% of participants reported financial stress related to diabetes, while 48% reported engaging in maladaptive cost-coping behaviors. This is similar to Patel et al. (43) in the United States, who reported that 59% and 39% of participants with uncontrolled diabetes reported financial toxicity and cost-related non-adherence, respectively. Although cost-related non-adherence is common among those with diabetes (44), this study provides evidence on how financial stress is significantly associated with engagement in maladaptive cost-coping strategies. Financial toxicity and cost-related non-adherence were not associated with glycemic control. Although cost-related non-adherence was significantly associated with glycemic control in Walker et al. (28), who similarly reported that financial strain is not associated with glycemic control. This is contrary to Patel et al. (21), who found a significant association between financial toxicity and glycemic control. In the current study, the possible reason might be that the report on financial toxicity is personal and participants responded based on their perceived financial stress and excludes the financial support received from children, friends, and family, which is prevalent in a collectivistic culture; hence, this financial support might have masked the potential effect. This interpretation is further supported by the qualitative theme of “expensive, but managing with financial support”. One participant with worse financial toxicity reported that: “It is taking a lot of money from me”, while also acknowledging that children provided financial support for medications: “It is the children. They send the money for drug every month” [sic]. Additionally, the theme “diet control to reduce money spent on drugs” revealed that some patients with worse financial toxicity and good glycemic control have adopted dietary control strategies to reduce medication costs. One of the participants stated: “That is why I controlled my food because I know that I don’t have money to control my diabetes.” In contrast, those with worse financial toxicity and poor glycemic control cited that financial constraints limited adherence to both medication and dietary recommendations.

Moreover, age, number of medication, use of insulin, and study center were consistently significantly associated with glycemic control in all the models. The participants aged <55 years were more likely to have poor glycemic control. Although Ibrahim et al. found that older age is associated with good glycemic control (13), our finding is consistent with other studies that reported higher odds of poor glycemic control in younger adults (45–47). The participants with early onset of T2DM might struggle with adaptation to the disease and treatment plan, as it might be difficult to manage a chronic condition with other life struggles. Elderly population might have adapted due to longer disease duration, experience of chronic disease management, and management support from children. Early management support for younger adults could help delay most microvascular complications (12), prolong life expectancy, and improve quality of life (48).

Paradoxically, there was increased odds of poor glycemic control from using two, three or more diabetic medications compared to one. This is similar to David et al. (14), who found that those prescribed one diabetic medication had a higher odd of good glycemic control, and Mamo et al. (49) in Ethiopia, who found dual therapy to be associated with poor glycemic control. While this association may reflect more advanced disease severity or progressive disease requiring multiple medications, the possible reason might be drug-therapy problems. In Zazuli et al. (50), the number of medications significantly predicted (B: 0.50) the number of drug-related problems, including inappropriate drug selection and patient-reported side effects. Qualitative findings revealed that participants might have misconceptions about the negative effect of polypharmacy. Moreover, *post-hoc* analysis showed that increased number of drugs was significantly associated with ‘skipping doses to make medication last longer’, a maladaptive cost-coping behavior. Similarly, insulin use was associated with poor glycemic control. This is consistent with the findings by Egede et al. (51) in the United States, who found poor glycemic control was 3.53 higher in individuals using insulin combined with oral hypoglycemic medication. For instance, in the qualitative strand, while the physician increases the dose of insulin at every follow-up due to poor control, the patient self-adjusted due to fear and misconception of the effect of prolonged use on the kidney. Moreover, the physician was unaware of poor dietary habits and non-adherence to dosages, which was admitted during interview. A patient noted: “I am on insulin, as at yesterday, the doctor said I should be using 16. Even he pushed to about 18. I started from 14, 16 and yesterday he said I should be using 18. This yesterday night I didn’t use 18, I used 16. Because I asked him yesterday that what is the after effect if I use 18, he said there is no side effect, but I cannot believe him. So yesternight, I just gave myself the injection of 16. I used 16 yesterday, and I woke up well.” This finding highlights the need for a paradigm shift in the healthcare system. The need to add more drugs or introduction of insulin should be the starting point of intensive treatment and interprofessional monitoring. Instead of responding to poor glycemic control with addition or intensification of medication alone, it is important to explore other patient lifestyle barriers that may hinder the management the disease. Participants should be followed more closely, and care management should be streamlined. One of the participants noted: “There is nothing being taught here than drugs. It is only drugs. There is nothing. They will just say go and use your drugs.” “If we just come, the nurses will just do BP and blood sugar testing and tell us to see the doctor; that’s all they do. When we get to the doctor, they will just ask about our drug compliance, and we tell them. The other time they go further is when the blood sugar is high; at least if the blood sugar is high, it is only a day. [sic]”.

Moreover, study site B was significantly associated with poor glycemic control. This may be due to the unavailability of an endocrinologist at the center. This is consistent with the findings by Chan et al. (15), who reported that specialist care has an impact on glycemic control. A study in southeastern Nigeria found that practice78% of primary care physicians with 17 years of practice had never participated in diabetes training after graduation, and 80% were unaware of diabetes clinical practice guidelines (52). Hence, training of primary care physicians and improved access to specialist care, such as an endocrinologist, are essential.

### 4.1 Strength and limitation

This study employed an explanatory sequential design to comprehensively examine how psychosocial, behavioral, and financial factors are associated with poor glycemic control. The large sample size, inclusion of multiple centers, and use of HbA1c as a measure of long-term glycemic control strengthen the study with previous studies conducted in Nigeria. However, this study used a convenience sample of patients with type 2 diabetes attending follow-up clinics, which may not be representative of the broader diabetic population across the hospitals and other levels of care in the country. The questionnaires were self-reported, and subjectivity in self-reporting and out-patient clinic settings may have introduced social desirability reporting and bias. Moreover, there was no established cut-off for diabetes self-management and financial toxicity measures, and the use of median split is sample-dependent and may not represent the cut-off for other samples or diabetic populations.

## 5 Conclusion

Younger age, use of two or more diabetic medications, use of insulin, poor self-management, diabetes-related distress, and lack of endocrinologist availability at the study site were independently associated with poor glycemic control. Hence, the healthcare system should prioritize training of primary care physicians and inclusion of endocrinologists in diabetes care. Since physicians have only limited time during consultation at follow-up, trained diabetes care nurses at outpatient clinics may help provide diabetes education grounded in behavioral modification and psychosocial support. Future interventional studies should explore the effectiveness of nurse-led case management models in coordinating the care of patients with diabetes to improve glycemic control. Additionally, with the increasing prevalence of early-onset type 2 diabetes, younger adults may require sustained support beyond traditional acute-care disease approaches to chronic conditions.

## Data Availability

The raw data supporting the conclusions of this article will be made available by the authors, without undue reservation.
