# Supplementary material for: Glycemic control and association with diabetes-related distress, self-management behavior, financial toxicity, and cost-related non-adherence: a mixed-methods study
Source: Front Endocrinol (Lausanne). 2026 Jun 5;17:1857675. doi: 10.3389/fendo.2026.1857675 (PMC13278867; doi:10.3389/fendo.2026.1857675)
Supplement: Supplementary file 1 [file SupplementaryFile1.docx]

Interview guide

1. How does your financial situation affect your diabetes control?
2. Do you think diabetes-related distress affect your diabetes management or diabetes control?
3. How has your knowledge of self-management affected your diabetes management?
4. How do you manage the costs of living with diabetes (food, medication, diabetic supplies)?
5. How do you feel about the amount of money you have to pay out-of-pocket for diabetes?
6. What instrumental support do you consider essential in managing your condition (e.g. self-management education, coping with the emotional distress of diabetes, financial support such as insurance)?
7. What factors have been most influential in helping you manage your condition?
8. What other factors have influenced your management efforts?
